# Ultrasound-triggered herceptin liposomes for breast cancer therapy

**DOI:** 10.1038/s41598-021-86860-5

**Published:** 2021-04-06

**Authors:** Amal Elamir, Saniha Ajith, Nour Al Sawaftah, Waad Abuwatfa, Debasmita Mukhopadhyay, Vinod Paul, Mohammad H. Al-Sayah, Nahid Awad, Ghaleb A. Husseini

**Affiliations:** 1 Department of Chemical Engineering, American University of Sharjah, Sharjah, UAE; 2 Department of Biology, Chemistry and Environmental Sciences, American University of Sharjah, PO. Box 26666, Sharjah, UAE

**Keywords:** Biophysics, Cancer, Nanoscience and technology

## Abstract

The functionalization of liposomes with monoclonal antibodies is a potential strategy to increase the specificity of liposomes and reduce the side-effects associated with chemotherapeutic agents. The active targeting of the Human Epidermal growth factor Receptor 2 (HER2), which is overexpressed in HER2 positive breast cancer cells, can be achieved by coating liposomes with an anti-HER2 monoclonal antibody. In this study, we synthesized calcein and Doxorubicin-loaded immunoliposomes functionalized with the monoclonal antibody Trastuzumab (TRA). Both liposomes were characterized for their size, phospholipid content and antibody conjugation. Exposing the liposomes to low-frequency ultrasound (LFUS) triggered drug release which increased with the increase in power density. Trastuzumab conjugation resulted in enhancing the sensitivity of the liposomes to LFUS. Compared to the control liposomes, TRA-liposomes showed higher cellular toxicity and higher drug uptake by the HER2 + cell line (SKBR3) which was further improved following sonication with LFUS. Combining immunoliposomes with LFUS is a promising technique in the field of targeted drug delivery that can enhance efficiency and reduce the cytotoxicity of antineoplastic drugs.

## Introduction

Breast cancer is the most common type of cancer in females, constituting the second leading cause of death globally^1,2^. The treatment of breast cancer is complex and relies on several factors, including the type of tumor, tumor size, grade, proliferation rate and lymph node status. For early-stage breast cancer, surgery is usually recommended to remove the tumor and its resection margin. For more aggressive tumors, the treatment course may involve chemotherapy or hormonal therapy before surgery (referred to as neoadjuvant therapy). After surgery, the aim is to lower the risk of recurrence and to get rid of any remaining cancer cells in the body; therefore, adjuvant therapies are used. Adjuvant therapies may include radiation therapy, chemotherapy, hormonal therapy, immunotherapy and/or targeted therapy^3,4^. In recent years, the presence of breast cancer tumor markers has been investigated, and several markers were identified, including the estrogen receptor (ER), progesterone receptor (PR) and the human epidermal growth factor receptor 2 (HER2)^3,5^. Human epidermal growth factors belong to a family of transmembrane receptor tyrosine kinases (RTKs). HER2 is involved in important stages of growth and cell differentiation and is normally expressed at low levels in the epithelial cells of various organs such as the lungs, bladder, pancreas, breast, and prostate^6,7^. HER2 overexpression can lead to malignant progression, which justifies the unfavorable prognosis of breast, ovarian, gastric, and prostate cancers^3^. HER2 overexpression is present in approximately 25% of all breast cancers and is usually associated with more aggressive disease and endocrine therapy resistance. Studies showed that HER2 is uniformly distributed within the tumor and its expression induces significant apoptosis in breast cancer cells. These characteristics make it an appealing choice as a target in targeted therapy^3,6,7^.

The treatment of HER2-positive breast cancer is continuously evolving. One of the significant advancements in breast cancer treatment was the development of mAb-based therapies. Targeted therapy options for HER2-positive breast cancer include Pertuzumab (Perjeta), Trastuzumab (Herceptin), Tucatinib (Tukysa), Neratinib (Nerlynx), Margetuximab (Margenza), DS-8201 (Enhertu), and Ado-trastuzumab emtansine or T-DM1 (Kadcyla)^4^. The humanized monoclonal antibody Trastuzumab (TRA), sold under the brand name Herceptin among others, was FDA approved in 1998 for the treatment of HER2-positive breast cancers^8–10^. TRA can enhance the effects of chemotherapy and reduce the risk of recurrence when used as an adjuvant therapy^11–13^. This immunotherapeutic agent is a humanized IgG(1) kappa monoclonal antibody (145.5 kDa) with a high and specific affinity towards HER2 receptors^14^. TRA can prevent HER2 hetero-dimerization and stop tumor development cell signaling via several mechanisms, including reduced PI3K/Akt signaling, enhanced degradation of HER2 receptors, and antibody-dependent cellular cytotoxicity (ADCC). Hence, this drug is considered a HER2 receptor antagonist^15–17^.

As mentioned earlier, TRA can induce antitumor responses as a single agent and is currently used as a first-line treatment of breast cancer. However, drug resistance is inevitable, necessitating alternative treatment options^18,19^. Studies have reported that the efficacy of TRA, when given in conjunction with chemotherapy, was superior to its effectiveness when used alone ^20–22^. Anti-HER2 immunoliposomes combine the tumor-targeting properties of mAbs, with the drug delivery properties and multi-drug resistance (MDR) mitigation ability of sterically stabilized liposomes encapsulating chemotherapeutic agents; offering prolonged inhibition of the HER2 pathway across multiple lines of treatment which will hopefully result in continued improvements in outcomes for HER2-positive breast cancer patients^18,19,23^. 

Concerning novel cancer treatment methods, smart drug delivery systems (SDDSs) have gained prominence in the field of targeted drug delivery^24,25^. SDDSs are nanostructures capable of reducing drug side effects, increasing blood circulation time, and increasing drug concentrations at target sites, thus ensuring better patient compliance^26, 27^. SDDSs release their payloads at target sites in response to internal or external triggers such as temperature, pH, enzymes, light, mechanical waves (e.g., ultrasound) or magnetic fields^28–32^. Several nanostructured delivery systems have been studied for cancer treatment, such as liposomes, solid lipid nanoparticles, metal organic frameworks, micelles, carbon nanostructures, dendrimers, quantum dots and antibody–drug conjugates (ADCs)^33^.

Liposomes are one of the most widely used nanocarriers in drug delivery^34^. Liposomes are nanosized to microsized concentric spheres of phospholipid bilayers separated by aqueous compartments. Liposomes assume this bilayer conformation to shield the hydrophobic tails of phospholipids from aqueous environments^35,36^. Furthermore, liposomes can be functionalized by conjugating different moieties to their surfaces^37, 38^. For instance, the circulation-time of liposomes can be increased and their detection by the reticuloendothelial system (RES) reduced by conjugating stealth-imparting polymers to their surfaces^39–41^. The most widely used polymeric substance is poly-ethylene glycol (PEG). Another example of such modifications involves the conjugation of ligands to the surfaces of liposomes to enhance their selectivity and tumor targetability^42^. These ligands have a binding affinity toward receptors, which tend to be overexpressed on the surface of cancer cells^6,40^. Some commonly used targeting ligands include carbohydrates, proteins, aptamers, antibodies, and peptides^43^.

Antibody therapeutics have revolutionized the treatment of cancer over the past few decades. Antibodies are being used in targeted therapy to deliver potent chemotherapeutic agents in the form of antibody–drug conjugates (ADCs) or as targeting ligands decorating the surfaces of nanocarriers^44^. Immunoliposomes provide a complementary, and in certain aspects a more advantageous, drug delivery strategy to ADCs. The large size of ADCs (more than 150 kDa) often hinders their diffusion into the intratumoral space; studies have estimated that only between 0.003 and 0.08% of the injected ADC dose per gram of tumor will accumulate in the tumor^44,45^. Moreover, immunoliposomes have a higher drug-carrying capacity (20,000–150,000 drug molecules/liposome) compared to ADCs (because an excessive drug to antibody ratio can lead ADCs to aggregate and be cleared by plasma proteins)^46,47^.

As discussed above, once SDDSs reach their target sites, they release their contents when exposed to either an internal or external stimulus^30^. Several external triggers have been investigated; however, the trigger of choice in this work is ultrasound (US). US is a cyclic sinusoidal acoustic wave with frequencies higher than those of the human hearing range (> 20 kHz)^48^. US waves have a high-pressure phase (compression) at the upper peaks and a low-pressure phase (rarefaction) at the lower peaks^49,50^. In therapeutic applications, the biological effects of US can be either thermal or mechanical^51^. Thermal effects are caused by energy dissipation, while the mechanical effects occur because of the acoustic wave propagation and pressure variations^52,53^. The mechanical effects of US manifest as acoustic cavitation events, which is the formation of gas bubbles due to changes in pressure and is generally divided into stable and transient cavitation^51^. In stable cavitation, the bubble expands and contracts about an equilibrium radius; however, in transient cavitation, the bubble grows rapidly in size and eventually collapses violently. Cavitation depends on the intensity of US and occurs only when a certain threshold (determined using the mechanical index) is reached. The mechanical index (MI) is a parameter that quantifies the probability of transient cavitation happening and is calculated using the following Eq. ^6, 7,49^.

1 $${\varvec{MI}} = \frac{{{\varvec{P}}_{{{\varvec{neg}}}} }}{{\sqrt {\varvec{f}} }}\user2{ }$$

where *P*_*neg*_ is the negative pressure in MPa, and *f* is the frequency in MHz.

In this study, calcein and Doxorubicin (DOX)-loaded pegylated liposomes were synthesized and functionalized with the monoclonal antibody) TRA to target breast cancer cells. LFUS was then applied to trigger the release of calcein and DOX from the liposomes, thus enhancing their uptake by the cancer cells.

## Materials and methods

### Materials

Dipalmitoylphosphatidyl choline (DPPC) and 1,2-distearoyl-sn-glycero-3-phosphoethanolamine-N [amino (polyethylene glycol)-2000] (DSPE-PEG (2000)-NH2) were obtained from Avanti Polar Lipids Inc. (Alabaster, AL, USA, supplied by Labco LLC. Dubai, UAE). Cholesterol, calcein disodium salt, L-glutamine, antibiotic solutions (penicillin and streptomycin), trypsin, fetal bovine serum (FBS), RPMI-1640 medium, Dulbecco’s Phosphate Buffered Saline (DPBS) medium and the bicinchoninic acid (BCA) kit were obtained from Sigma Aldrich Chemie GmbH (Munich, Germany, supplied by Labco LLC. Dubai, UAE). Chloroform was obtained from Panreac Quimica S.A. (Spain). Doxorubicin-hydrochloride was obtained from Euroasian Transcontinental (Lower Parel, Mumbai, India). Sephadex G-100, Sephadex G-25, and Sephacryl S200 HR were obtained from Sigma-Aldrich (Munich, Germany, supplied by Labco LLC. Dubai, UAE). Trastuzumab (Herceptin) was obtained from Hoffmann-La Roche Limited (Basel, Switzerland, supplied by Aster pharmacy, Sharjah, UAE). 2,4,6 trichloro-1,3,5 triazine (cyanuric chloride) was obtained from Sigma-Aldrich (St. Louis, MO, US, supplied by LABCO LLC. Dubai, UAE). SKBR3 (HER2 + cells) and MDA-MB-231 (HER2- cells) were purchased from American Type Culture Collection (ATCC, Manassas, VA, USA).

### Preparation of control liposomes

Liposomes were prepared using the thin-film hydration method. Briefly, liposomes were prepared using cholesterol, DPPC, and DSPE-PEG(2000)-NH_2_ at molar ratios of 30:65:5, respectively. The lipids were dissolved in 4 ml chloroform in a round-bottom flask. The chloroform was then evaporated using a rotary evaporator under vacuum at 50 ºC for 15 min until a thin film was observed on the walls. Next, the lipid film was hydrated using 2 ml of a 30-mM calcein solution and the pH adjusted to 7.4. To obtain unilamellar vesicles, the solution was sonicated for 2 min using a 40-kHz sonicator bath (Elma D-78224, Melrose Park, IL, USA). Whereas for particle size reduction, liposomes were extruded using 200-nm polycarbonate filters (Avanti Polar Lipids, Inc., Alabaster, AL, USA). The purification of the formulation was performed using a Sephadex G-100 column (after equilibrating it with a borate buffer pH 8.5). Finally, the collected fractions were stored at 4 °C^6^.

The same protocol described above was used to prepare liposomes encapsulating ammonium sulfate instead of calcein. The ammonium sulfate method was used to load DOX into the formed liposomes. A 0.11-M solution of ammonium sulfate at a pH of 5.5 was prepared to hydrate the dry lipid film. Liposomes were purified with a Sephadex G-25 gel filtration column previously equilibrated with HEPES buffer (0.26 M Sucrose, 0.005 M ascorbic acid and 0.016 M HEPES). Next, DOX was added to the liposomal solution providing a DOX to lipid ratio of 1:6 (w/w). The liposome-DOX solution was kept in a water bath at 60℃ for 45 min with mild stirring. The resulting solution was then centrifuged through a Sephadex G-25 gel filtration column equilibrated with PBS (pH ~ 7.4)^7^.

### Preparation of Trastuzumab (TRA)-liposomes

The functionalization of the liposomes with TRA was performed using a double substitution reaction. Calcein and DOX liposomes were prepared separately using the procedures detailed in the previous section. As for the functionalization procedure, cyanuric chloride was dissolved in acetone to make a 10 mg/ml solution, of which 9.23 μl was diluted in 0.5 ml deionized water. This solution was then added to 1 ml of the liposomal formulations. The reaction was conducted at a pH of 8.5 and 0 °C and was left to stir for 3 h, allowing the nucleophilic substitution of the chloride particle on the cyanuric chloride with the proton of the NH_2_ group of the liposomes. The second reaction involves the linking of the N-terminus on the amino acids of TRA to cyanuric chloride. One milligram of TRA was dissolved in 0.5 ml borate buffer (pH 8.5), this solution was then added to the liposomes, and the reaction was kept stirring overnight. Finally, excess TRA and any free drugs were purified in a Sephacryl S-200 h column equilibrated with PBS (pH of 7.4). The liposomes were collected and stored at 4 ºC^6,7^.

### Particle size and polydispersity evaluation

The particle size and polydispersity index of liposomes and immunoliposomes were measured at 25 °C. The intensity-weighted hydrodynamic radius was determined using DynaPro NanoStar (Wyatt Technology Corp., Santa Barbara, CA, USA).

### Estimation of phospholipid content

The phospholipid content of liposomes was determined using the Stewart Assay. The mixing of a phospholipid-containing chloroform solution with ammonium ferrothiocyanate at room temperature yields a colored complex that partitions in the chloroform phase and whose maximal absorbance is 485 nm. The liposome samples were dried under vacuum and then dissolved in chloroform. This solution was then sonicated to break the liposomes to their constituent lipids. The liposomes-chloroform solution was transferred to a centrifuge tube where 2 ml of ammonium ferrothiocyanate was added. The centrifugation step resulted in a biphasic system; the top dark layer was removed and discarded, while the bottom clear chloroform layer was transferred to a quartz cuvette, and its optical density measured using ultraviolet–visible (UV–Vis) spectroscopy at A_max_ = 485 nm against chloroform as a blank. An average of the six measurements was taken.

### Trastuzumab conjugation to the liposomes

TRA conjugation efficiency to liposomes was determined by the Bicinchoninic Acid Assay (BCA). The BCA reagent was prepared by mixing QuantiPro QA buffer, QuantiPro QB, and CuSO4 in a ratio of 25:25:1, respectively. One milliliter of the reagent was added to 1 ml of PBS and 100 μl of the liposomal solution, followed by incubation at 60ºC for 1 h. The optical density of the samples was measured using UV–Vis spectroscopy at 562 nm. Moreover, the molecular weight of TRA and DPPC, in addition to the results obtained from the BCA and Stewart assay, were used to determine the number of TRA molecules per vesicle.

### Power density measurements

The ultrasonic probe used in this study (model VCX750, Sonics & Materials Inc., Newtown, CT) produced LFUS (at 20 kHz). On the display of the 20-kHz system, you can dial a power amplitude as a percentage (in our experiments, we selected amplitudes of 20%, 25% and 30%). Measuring the power densities corresponding to these three amplitudes was conducted using a hydrophone (Bruel & Kjaer 8103, Nærum, Denmark). The tip of the probe was placed inside the center of a water bath (2 cm from the surface) while the hydrophone was inserted inside the water bath at a constant depth (3 cm from the probe). Upon US application, the waves produced by the US create a pressure variation which can be detected by the hydrophone and converted into voltage signals. The signals are then fed to a digital storage oscilloscope (Tektronix TDS 2002B) and later analyzed using the MATLAB software. A 2D map was created by keeping the hydrophone at a constant distance in the ‘Z’ direction (the depth of the hydrophone), while raster scanning was performed in an area of 7 cm × 5 cm (in X–Y direction) around the probe, and the signals were picked up at an interval of 1 cm. The measurements were completed for all three amplitudes (20%, 25% and 30%). The measured voltage signals were then converted into acoustic pressure in Pascal using the equation:

2 $$P = \frac{{Vrms \,\left( {\varvec{V}} \right)}}{{Voltage\, Sensitivity\left( {\frac{{\user2{\mu V}}}{{{\varvec{Pa}}}}} \right)}}$$

The value of the hydrophone voltage sensitivity was provided/reported by the hydrophone manufacturer as 30 µV/Pa.

And the ultrasound power density ‘I’, in Watt/cm^2^, is given by the equation:

3 $$Power Density, I = \frac{{P^{2} }}{Z}$$

where ‘Z’ is the acoustic impedance of the medium (1.48 × 10^6^ kg.m-2.S-1, the impedance of water) and ‘P’ is the pressure measured in Pascals.

### Low-Frequency ultrasound release studies

The release of calcein and DOX from liposomes was triggered using a 20-kHz low-frequency ultrasonic probe (model VCX750, Sonics & Materials Inc., Newtown, CT) and monitored by fluorescence changes using a QuantaMaster QM 30 Phosphorescence Spectrofluorometer (Photon Technology International, Edison NJ, USA). Calcein is a fluorescent molecule with excitation and emission wavelengths of 495 and 515 nm, respectively. As for DOX, the emission wavelength is 595 nm, while its excitation wavelength is around 485 nm. The sample to be tested was prepared by diluting 75 μL of liposomes in 3 mL of PBS in a fluorescence cuvette. The initial fluorescence intensity *I*_*o*_ was measured for 60 s before sonication. Then, US was applied in a pulsed mode, with 20 s *on* and 10 s *off* for calcein liposomes and 20 s *on*, 20 s *off* for DOX-liposomes to account for the thermal effects of US. The release was performed using three different power densities, 6.2, 9 and 10 mW/cm^2^. The pulsed US cycles mentioned earlier were continued until a fluorescence plateau was reached, at which point 50 μL of Triton X-100 (Tx100) were added to the sample to lyse the liposomes and release all their encapsulated contents, simulating 100% drug release. The percent of drug released from the liposomes was represented by the Cumulative Fraction Release (CFR), as follows:

4 $$CFR = \frac{{I_{t} - I_{o} }}{{I_{\infty } - I_{o} }}$$

where *I*_*0*_ represents the baseline intensity, *I*_*t*_ represent the intensity at time, *t*, and $I_{\infty }$ represents the highest fluorescence intensity value obtained.

### MTT assay

SKBR3 (HER2 + cells) and MDA-MB-231 (HER2- cells) were cultured in DMEM and RPMI media, respectively, supplemented with 10% Fetal Bovine Serum and 1% penicillin/streptomycin. Cells were grown at 37 °C under 5% CO_2_. Aliquots containing 1 × 10^4^ cells per well were seeded into 96-well plates and incubated for 24 h prior to treatment to ensure proper cellular confluency. After 24 h, different treatments were added in triplicates to the cells with a concentration of 8 µM per well. The cells were treated with free DOX, DOX-loaded control liposomes and DOX-TRA liposomes. Drug-free control liposomes (liposomes encapsulating PBS buffer) were also added as a negative control. The cells were then incubated with the different liposomal formulations for 4 h. Following incubation, the plates were subjected to continuous LFUS for 5 min in a 35-kHz bath. One plate of each cell line was not subjected to LFUS to serve as a control. The plates were then returned to the previous incubation conditions for 48 h. Following the incubation period, the cell culture medium was replaced with the MTT medium, containing 20 μl of the sterile MTT dye (5 mg/ml), and further incubated at 37 °C for 4 h. After the incubation, 100 μl of DMSO (Sigma-Aldrich, USA) was mixed with the medium and incubated for 15 min. Finally, absorbance values were read using a microplate reader (AccuReader, Nangang, Taipei, Metertech, Taiwan) at 570 nm. For each group, three replicates were analyzed, and a mean value was estimated. The cell viability was calculated by dividing the optical density (OD) value of the experimental group by the mean OD of the control group and multiplying it by 100%.

### Flow cytometry analysis

For the flow cytometry assay, both SKBR3 and MDA-MB-231 cells (2 × 10^5^ cells/ml) were seeded in 6-well plates for 24 h. On the following day, the cells were treated with the control and TRA-DOX liposomes and further incubated for 4 h, after which the cells were sonicated in a LFUS bath at a frequency of 35-kHz for 5 min and a power density of 20 mW/cm^2^. One plate from each cell line was treated with both types of liposomes but was not exposed to LFUS to serve as a reference. Following sonication, the cells were again incubated for 1 h. Trypsin was then added to the plates to detach the cells, which were resuspended in PBS to prepare them for the flow cytometer analysis.

### Fluorescent microscopy imaging

SKBR3 cells (2 × 10^5^ cells/ml) were seeded in two 6-well plates for 24 h followed by incubation with either control or TRA-liposomes for 4 h. One of the two plates was sonicated using a sonicating bath (35 kHz) for 5 min. The media was removed from the wells, and the cells were washed with PBS before being fixed with 4% Formaldehyde. Further washing was carried out using PBS buffer before imaging. The plates were then examined using a fluorescent microscope (Olympus IX53, excitation filter at 470–495 nm and emissions at 510–550 nm).

### Statistical analysis

Results were reported as average ± standard deviation (SD). One-way ANOVA tests were used to compare the sizes of the control and immunoliposomes, while two-factor ANOVA tests were employed to analyze the LFUS release results. Both types of ANOVA tests assumed that both populations have similar variances, and two values are considered statistically different if *p* < 0.05 and if F < F_critical_ (unless otherwise stated).

## Results

### Liposomes characterization

Dynamic light scattering (DLS) measurements were performed to ensure the formation of liposomes and to measure the size of both types of synthesized liposomes. A polydispersity (Pd) index upper limit of 20% is generally acceptable for DLS measurements. Table 1 summarizes the average sizes of the three batches of liposomes with their respective standard deviations. Based on these findings, both types of liposomes (both calcein and DOX-loaded) are well within the size range for the EPR effects to take place.

**Table 1:** Size and polydispersity of Calcein and DOX-loaded control and immunoliposomes.

Liposomes	Radius (nm)		pd%	
	Calcein	DOX	Calcein	DOX
Control liposomes	89.54 ± 0.50	91.2 ± 1.47	11.28 ± 1.11	12.3 ± 3.01
TRA-liposomes	101.10 ± 1.13	94.9 ± 1.29	17.22 ± 2.34	11.2 ± 0.55

A one-way ANOVA analysis was conducted to compare the radii of the control liposomes and the immunoliposomes. The results showed a higher F-value compared to F_critical_, as well as a p-value lower than the standard alpha value of 0.05, which indicated that the two types of nanoparticles had statistically different radii.

**Figure 1:**
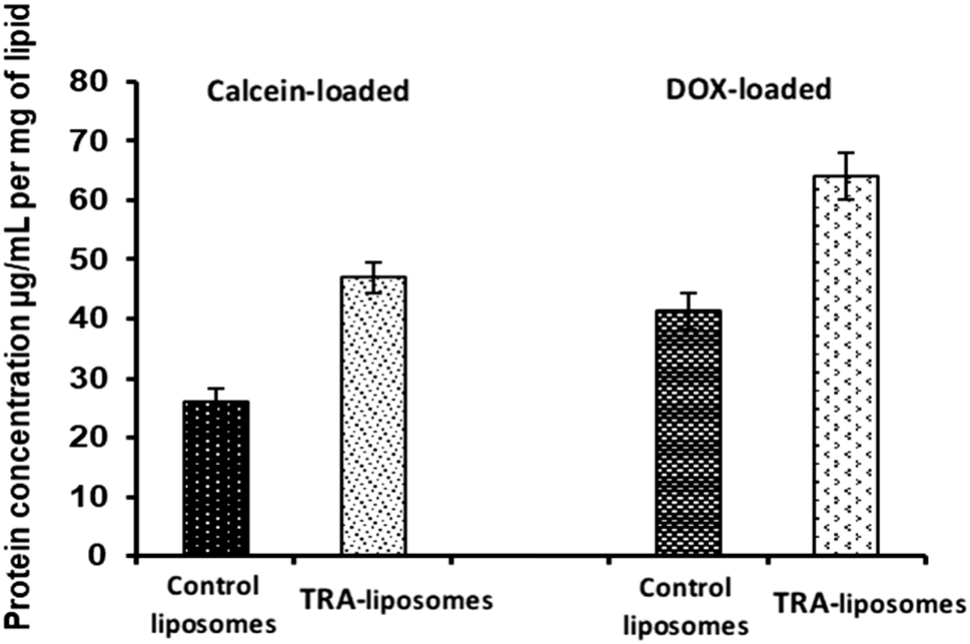
Protein concentrations (per mg of lipids) for the control and TRA-liposomes in both calcein and DOX-loaded liposome.

To confirm the conjugation of TRA to liposomes, the results from the BCA and Stewart assays were used. As mentioned above, the BCA assay was used to determine the protein concentration in the samples in μg/ml, while the Stewart assay was used to determine the DPPC concentration in mg/l. According to the BCA results, TRA-liposomes exhibited a 1.5-fold increase in protein concentration compared to control liposomes for both calcein- and DOX-loaded liposomes (Fig. 1). Combining the findings of both assays yielded a weight-to-weight (w/w) ratio of proteins to lipids in μg/mg. The results of three batches were averaged to confirm conjugation consistency and calculate the standard deviation. Therefore the average number of lipid molecules constructing a single liposome vesicle would be 80,000^54^. Knowing the concentration of TRA and the lipids making up the liposomes, as well as their respective molecular weights, we estimated that approximately 9 TRA molecules were conjugated per liposome which is considered well within the optimal range necessary to induce sufficient cells cytotoxicity^55^.

### Determination of the different power densities of the LFUS

The power densities corresponding to the three investigated amplitudes produced by the LFUS processor were accurately measured using a 2D acoustic map in a water bath using a hydrophone. The produced map was analyzed to calculate the correct power density delivered by the probe to the samples. The new calculated power density values were 6.2. 9 and 10 mW/cm^2^ corresponding to amplitudes of 20%, 25%, and 30%. Figure 1 (Supplementary Materials) shows the 2D acoustic map of the power density values at various distances from the probe.

### Low-frequency ultrasound (LFUS) triggered release

**Figure 2:**
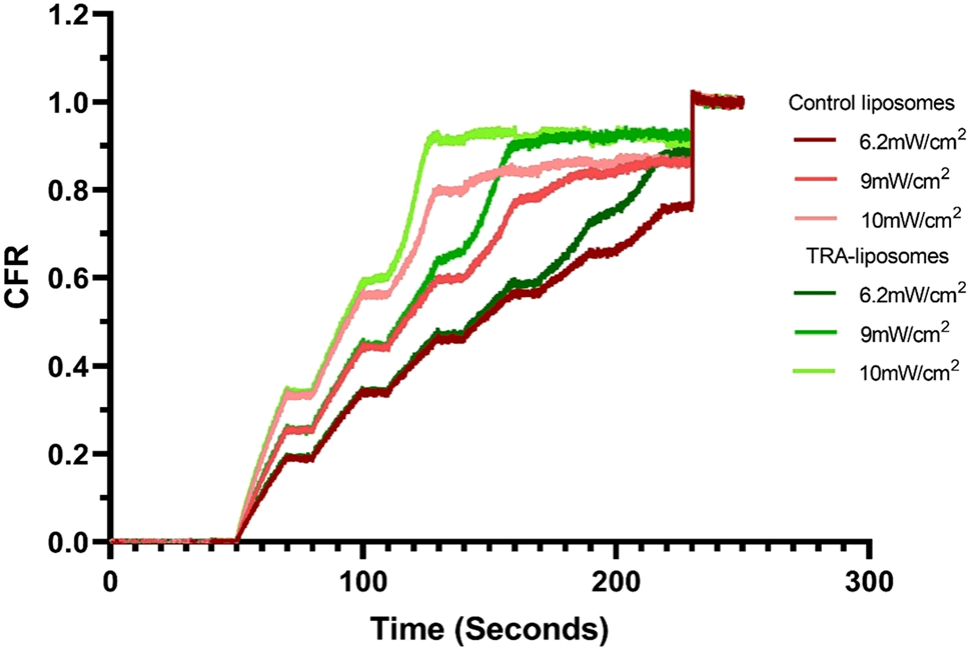
Normalized release profiles of the control and TRA-liposomes loaded with calcein at 6.2 mW/cm^2^, 9 mW/cm^2^ and 10 mW/cm^2^. Results are the average of three batches of liposomes with three replicates each.

LFUS release was performed on three batches of calcein-loaded control liposomes and immunoliposomes (TRA-liposomes). The release trends for the control liposomes are depicted in Fig. 2 and Table 2 which show the normalized online release rate of calcein from both types of liposomes. Generally, both types of liposomes demonstrated what could be considered “ideal” release profiles for controlled drug release systems. As seen in Fig. 2, as the power density increases, the release rate becomes steeper in both the control and TRA liposomes. This is expected due to an increase in cavitation events with increased power density.

**Table 2:** Summary of release results for calcein liposomes.

Liposome type	Power Density (mW/cm^2^)	CFR at plateau
Control liposomes	6.2	0.8530
	9	0.8820
	10	0.8554
TRA-liposomes	6.2	0.9109
	9	0.9257
	10	0.9204

Overall, TRA-liposomes were more acoustically sensitive, releasing around 92% of the fluorescent model drug, compared to approximately 86% released from the control liposomes following the sonication with the highest power density used (10 mW/cm^2^) as seen in Table 2 and Fig. 2. We attribute this behavior to the presence of the TRA molecules on the surface of these liposomes. This appears to slightly destabilize the membrane making the liposomes more susceptible to acoustic mechanical waves. The observed enhanced sensitivity of immunoliposomes to LFUS waves is advantageous because therapeutic drug levels could be reached in shorter exposure times.

Finally, the mechanical index (MI), is a parameter used to indicate the possibility of the occurrence of cavitation. The negative pressure (expressed in units of MPa) in the above equation is dependent upon the acoustic impedance of water, Z, and the intensity of the LFUS, I, (expressed in W/cm^2^). Negative pressure is denoted by Eq. (3).

5 $$P_{neg} = \sqrt {2 I Z}$$

Since the acoustic impedance of soft body tissues is comparable to that of water; when determining P_neg_, the acoustic impedance of water, i.e., 1.48 MPa sec/m was used^56,57^. The LFUS power densities used in this research were 6.2, 9, and 10 mW/cm^2^, corresponding to MI values of 0.096, 0.115, and 0.121, respectively. The threshold of collapse cavitation is expected to occur at around MI = 0.3, biological effects are observed at MI > 0.7, and tissue damage is expected to occur at MI > 1^58–61^. Therefore, the MI values used in this study are well below the collapse cavitation threshold of 0.3, indicating the occurrence of stable cavitation.

### Flow cytometry analysis of cellular uptake of calcein

**Figure 3:**
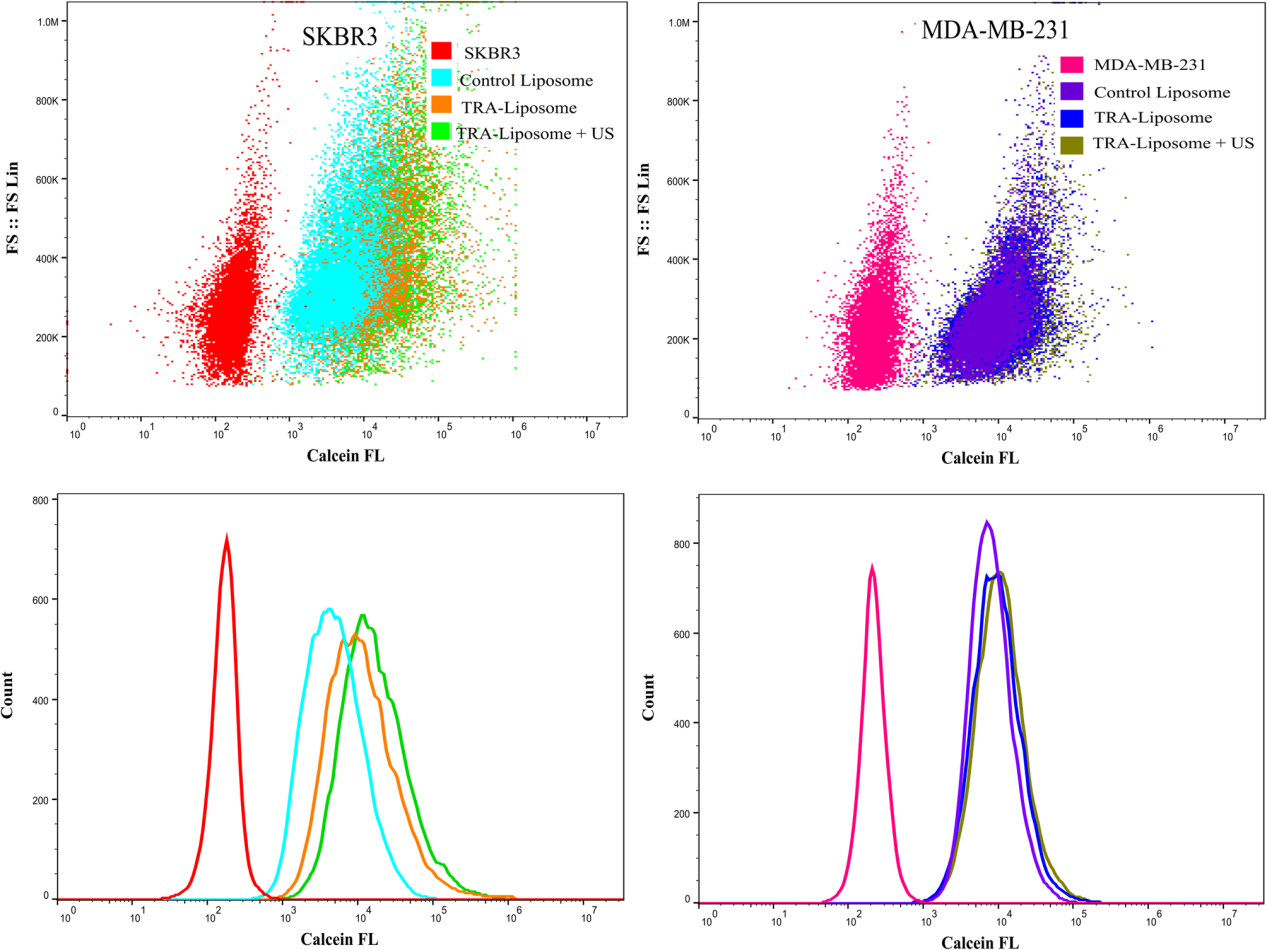
Flow cytometry analysis of calcein uptake by SKBR3 and MDA-MB-231 cells following their incubation with control liposomes or TRA-liposomes followed by ultrasound (US) sonication (35-kHz) for 5 min at a power density of 20 mW/cm^2^. Untreated cells served as a negative control for background fluorescence. An average of 1 × 10^4^ cells was analyzed from each sample with calcein fluorescence intensity showed on a four-decade log scale.

Flow cytometry analysis was carried out to determine calcein uptake by both the HER2 + (SKBR3) as well as HER2- (MDA-MB-231) cells when incubated with either the control or TRA-liposomes for 4 h. As shown in Fig. 3, the average calcein fluorescence intensity in SKBR3 cells significantly increased when incubated with TRA-liposomes (25,160) compared to the control liposomes (7236). Sonicating the cells with LFUS (35-kHz) for 5 min resulted in a further increase in calcein fluorescent intensity in the cells incubated with TRA-liposomes from 25,160 to 32,735. The comparison between the fluorescence intensity in the cells treated with TRA-liposomes  and subjected to US compared to those incubated with the control liposomes showed a 4.5-fold increase (from 7236 A.U.  to 32,735 A.U.).

The recorded increase in the fluorescence intensity of calcein inside SKBR3 cells when incubated with TRA-liposomes compared to control liposomes could be attributed to the higher uptake of liposomes due to the binding of TRA-liposomes to the overexpressed HER2 receptors. To test this proposed mechanism, the same experiment was repeated using a triple-negative cell line (MDA-MB-231). The results showed that no significant difference was recorded in calcein fluorescence intensity inside MDA-MB-231 cells incubated with either the control or TRA-liposomes showing fluorescence intensities of 10,022 and 13,914, respectively. This indicated that the low expression of HER2 receptors on the surface of MDA-MB-231 cells resulted in reducing the targeting ability of the TRA-liposomes and thus, low cellular uptake of the calcein encapsulated inside the targeted liposomes through receptor-mediated endocytosis.

**Figure 4:**
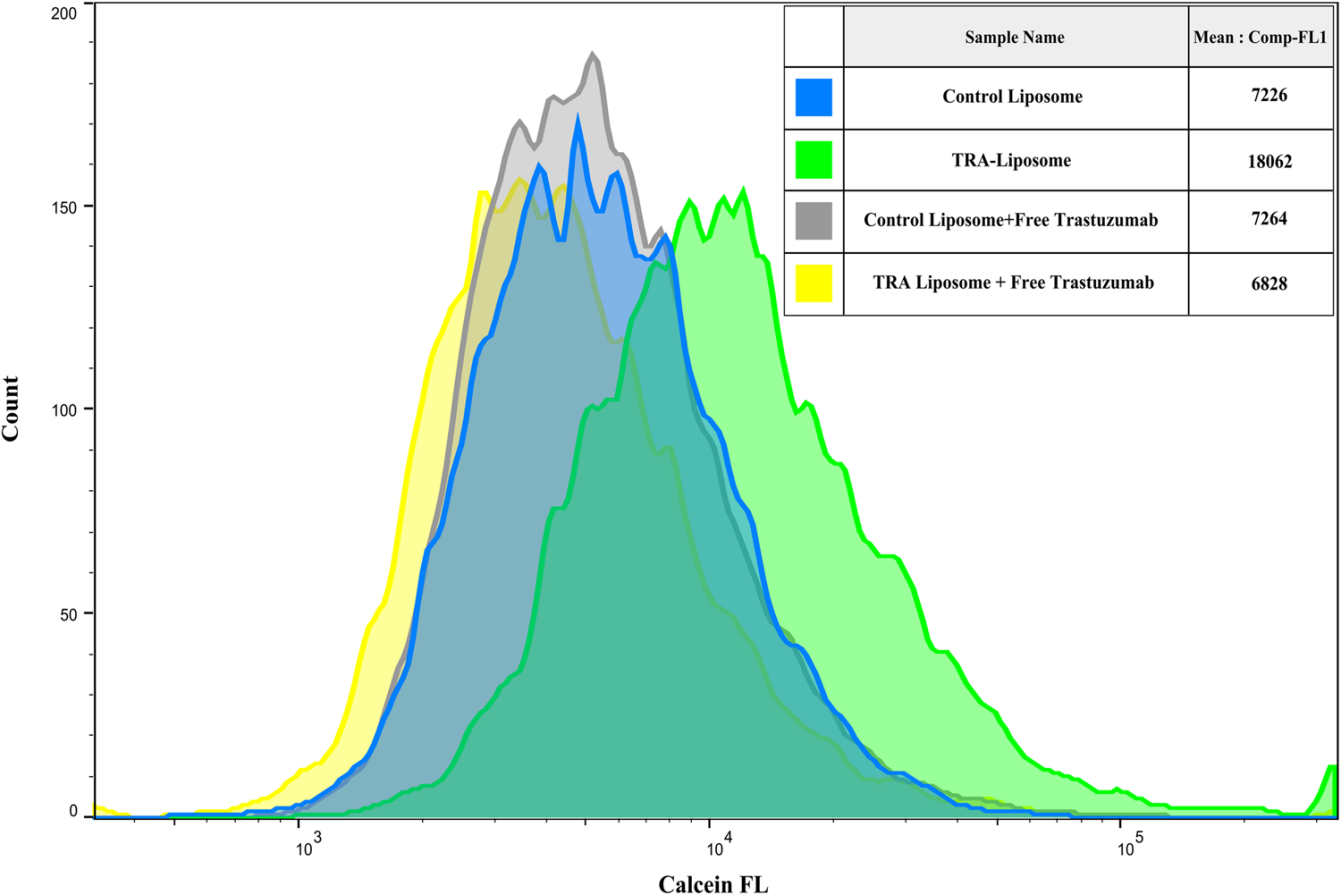
Flow cytometry analysis showing calcein fluorescence intensity inside SKBR3 cells incubated with the either the control or TRA-liposomes with or without prior incubation with free Trastuzumab (1 mg/ml). An average of 1 × 10^4^ cells was analyzed from each sample with calcein fluorescence intensity showing on a four-decade log scale.

The suggested mechanism of the binding of immunoliposomes to HER2 receptors overexpressed on the surface of SKB3 cells and the subsequent receptor-mediated endocytosis was examined. This was achieved by blocking HER2 receptors to prevent the targeted liposomes from binding to those receptors (i.e., competitive inhibitors), thus reducing the uptake of TRA-liposomes by the cells. SKBR3 cells were incubated with “control” liposomes and TRA-liposomes, calcein fluorescence present in the cytoplasm was detected using flow cytometry analysis. This was compared with SKBR3 cells incubated first with free Trastuzumab (1 mg/ml) for 30 min to allow it to bind to HER2 receptors prior to the addition of the liposomes. The cells were then incubated for 4 additional hours with the different liposomal formulations. As seen in Fig. 4, while no significant difference was recorded in calcein fluorescence values in the cells incubated with the control liposomes in the presence or absence of free TRA (p-value = 0.095), calcein uptake was significantly hindered when HER2 receptors were first blocked with free TRA (*p*-value = 3.23 × 10–5).

### Fluorescent microscopic images

**Figure 5:**
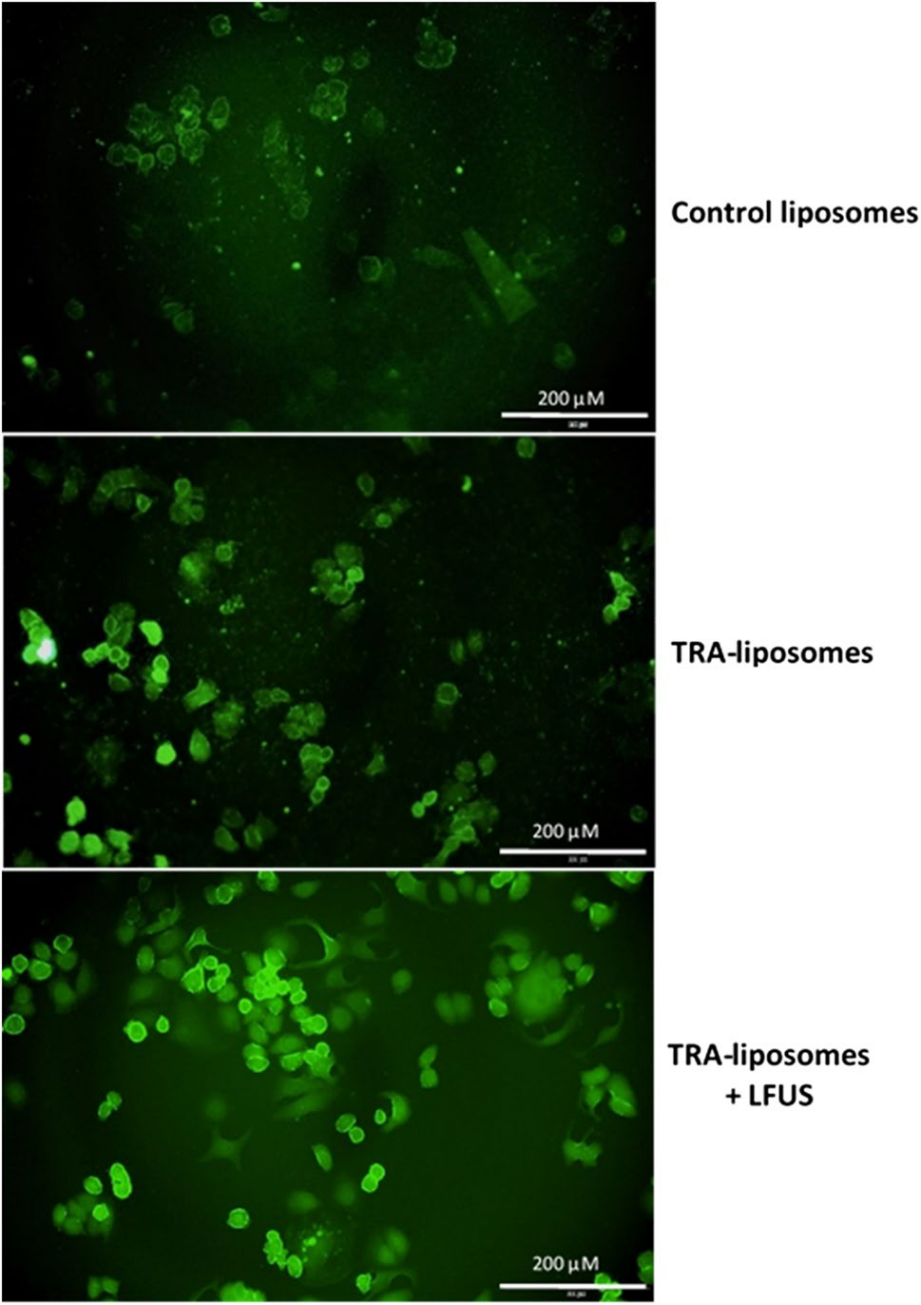
Fluorescence microscopy images of SKBR3 cells following 4 h incubation with control and TRA-liposomes encapsulating calcein in addition to TRA-liposomes exposed to LFUS (35-kHz) for 5 min.

Further visualization experiments were carried out to study the internalization of the control and targeted liposomes inside the SKBR3 cells. Figure 5 shows fluorescent microscopic images of SKBR3 cells following 4 h of incubation with either control or TRA-liposomes encapsulating the fluorescent model drug “calcein”. These images showed that the fluorescent dye was present in the cytoplasm of the SKBR3 cells. The intensity of calcein’s fluorescence was clearly higher in SKBR3 cells incubated with TRA-liposomes for 4 h compared to those incubated with the control liposomes for the same period. These findings support the previously obtained flow cytometry results and indicate that the recorded increase in calcein uptake from the immunoliposomes is due to the presence of the targeting ligand (TRA molecules) on the surface of these liposomes. This will enable their uptake by the cells through receptor-mediated endocytosis. Also, Fig. 5 shows that sonication with LFUS resulted in further enhancement of calcein fluorescence intensity. This can be caused by US triggering calcein release from the immunoliposomes.

### In vitro cytotoxicity analysis

**Figure 6:**
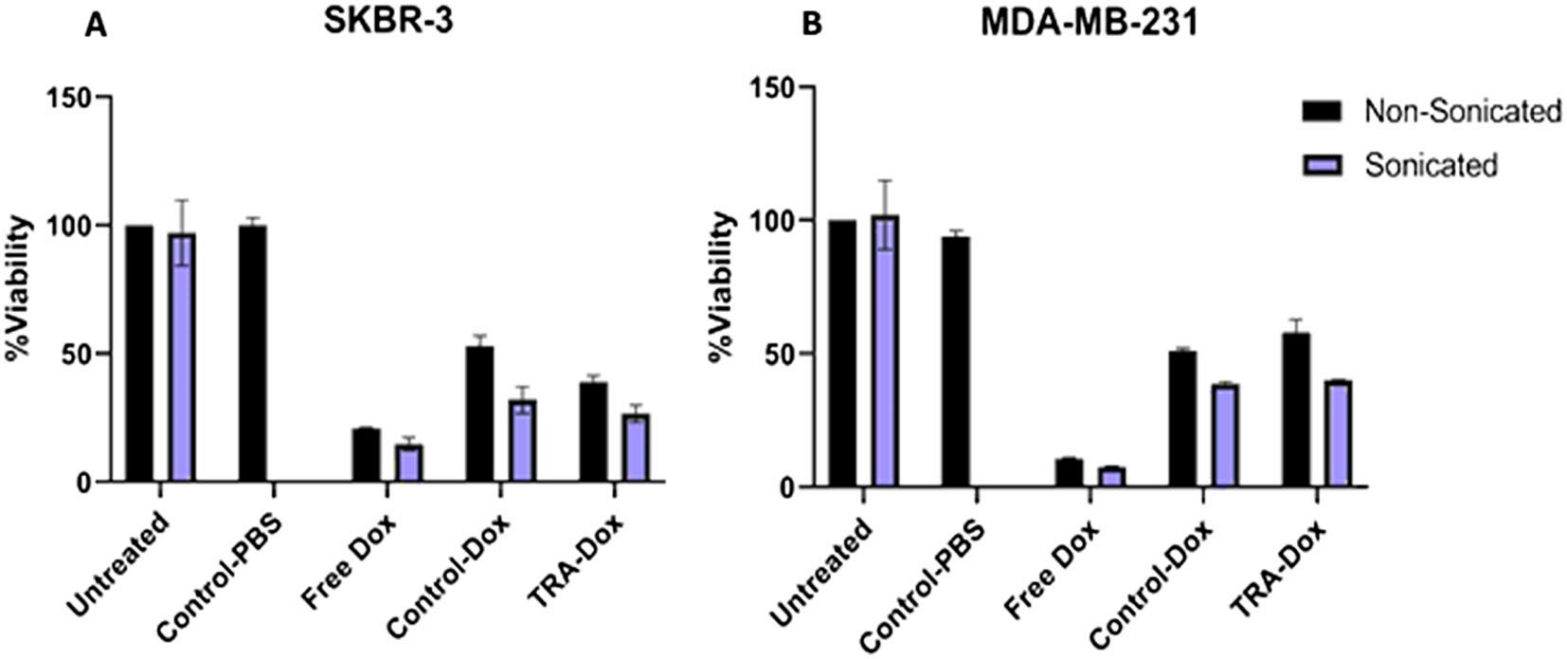
MTT results for (A) SKBR3 cells and (B) MDA-MB-231 cells treated with free DOX, Control-DOX liposomes and TRA-DOX liposomes (DOX concentration: 8 μM) in the absence and presence of LFUS exposure. Sonicated cells were exposed to continuous LFUS in a 35-kHz water bath for 5 min. Data are representative of three independent experiments (mean ± SD %, n = 3).

To assess the therapeutic efficiency of TRA-liposomes encapsulating DOX, the MTT assay was performed using both SKBR3 (HER2 +) and MDA-MB-231 (HER2-) breast cancer cell lines. As shown in Fig. 6, liposomes without DOX encapsulation were used as control and showed no significant effect on cell viability (~ 100%), which confirms that any recorded cytotoxicity is due to the action of DOX and not the liposomes themselves. Also, the toxicity of LFUS on the cells was also examined; the results showed that sonicating the cells with LFUS (35 kHz) had no significant effect on cell viability compared to the non-sonicated cells, as shown in Fig. 6 as well as Tables 1 and 2 (in the Supplementary Materials file).

Figure 6A shows the cell viability of SKBR3 cells following the incubation with different liposomal formulations. TRA-liposomes showed significantly higher toxicity compared to the control liposomes, i.e., 38.9 ± 2.54% and 53.1 ± 3.89%, respectively (p-value = 4.12 × 10–2). Upon exposure to LFUS, a further statistically significant reduction in cell viability was observed in TRA-liposomes treated cells from 38.9 ± 2.54% to 27.4 ± 0.260% (p-value = 3.18 × 10–3). Moreover, the cell viability of the cells treated with control liposomes also showed a statistically significant drop in cell viability from 53.1 ± 3.89% to 32.9 ± 1.31% (p-value = 1.08 × 10–2). These findings highlight the synergistic effects of using LFUS to trigger drug release from immunoliposomes targeting breast cancer. Additionally, DOX cytotoxicity increased when cells were sonicated compared to incubation with free DOX (8 μM) (viabilities of 14.8 ± 2.52% and 20.8 ± 0.5%, respectively, p-value = 2.93 × 10–2). This can be attributed to the sonoporation effect, which resulted in enhancing the cytotoxic performance of the free drug.

To validate the comparison and emphasize the effects of both LFUS exposure and active targeting of the immunoliposomes, Fig. 6B shows the cell viability of MDA-MB-231 (HER2- cell line) in response to the different treatments. No statistically significant difference was observed when MDA-MB-231 cells were treated with DOX-loaded TRA-liposomes and DOX-loaded control liposomes (58.0 ± 1.91% and 51.1 ± 0.933%, respectively, p-value = 1.24 × 10^–1^). Sonication with LFUS enhanced the cytotoxicity of both liposomal formulations with cell viability values of 40.1 ± 0.216% and 38.7 ± 0.858% for the TRA-liposomes and the control liposomes, respectively (p-value = 9.42 × 10^–2^).

The results show that combining TRA-liposomes with LFUS exhibited significantly higher drug toxicity in SKBR3 (HER2 +) cells compared to MDA-MB-231 (HER2-) cells showing cell viability values of 27.4 ± 0.260% and 40.1 ± 0.216% respectively (p-value = 7.53 × 10^–7^). Further analysis of the effect of increasing the incubation time of the cancer cells with the different liposomal formulations on both the cellular uptake and cell viability together with optimizing the different ultrasonic parameters such as frequency, power density, pulse duration, liposomal concentration, etc. is essential to further improve the benefits of this therapeutic platform.

## Discussion

Targeted drug delivery is a promising technique in cancer treatment that aims to deliver therapeutic drugs to the diseased site while reducing their contact with the healthy cells. Targeted nanocarriers are attracting increasing attention due to the variety in their synthesis and material properties. Liposomes are among the most successful nanocarriers due to their high biodegradability, biocompatibility, and ability to encapsulate both hydrophilic and hydrophobic drugs. Moieties can be easily conjugated to their surfaces to target different receptors overexpressed on the surface of cancer cells. Antibodies are an attractive choice as targeting molecules conjugated to liposomes (immunoliposomes) to target certain receptors, such as HER2 receptors, overexpressed on the surface of breast cancer cells. In this study, we are investigating the effect of conjugating a monoclonal antibody (trastuzumab) to pegylated liposomes and triggered with low-frequency ultrasound (LFUS) to enhance the release of the encapsulated drug in a spatially and temporally controlled manner.

Immunoliposomes are similar to other antibody–drug conjugates (ADCs) in terms of the antibodies conjugation to toxic drugs to target specific receptors on the HER2 positive breast cancers. This will enhance the efficiency and reduce the off-target toxicity of the antineoplastic agent. Despite their similarities, immunoliposomes use different strategies or concepts compared to that of ADCs’ but aim to achieve the same goals. Immunoliposomes are not only designed to bind to the target but also to be taken up by the cancer cell through receptor-mediated endocytosis. This allows a higher and more toxic concentration of one or more drugs to be delivered to the cells. Also, immunoliposomes can be employed to deliver large molecules such as genes.

Liposome-encapsulated doxorubicin was developed to reduce the toxicity while enhancing the targeting efficiency of DOX. These liposomes are surrounded by the polymer “polyethylene glycol (PEG)” which creates a hydrophilic shield that protects the liposomes from elimination and ensures a prolonged circulation time in the body. This, together with these liposomes' ability to extravasate through the leaky vessels surrounding the tumor, allow them to accumulate inside malignant tissues. Generally, DOX encapsulation inside the liposomes changes the pharmacokinetics of the drug and thus, results in a different cellular response and tolerability. Liposomal DOX has been studied widely over the past several years. Some liposomal formulations of DOX have been developed and extensively evaluated for their safety and efficacy. The two most advanced formulations are Doxil (used in the US since 1995) and Myocet (community authorization by the European Commission since 2000). Liposomal DOX is safer and showed similar or higher therapeutic efficiency while causing less cardiotoxicity and gastrointestinal toxicity compared to the conventional DOX in the treatment of metastatic cancers^62–68^.

Our synthesized immunoliposomes (TRA-liposomes) were within the recommended size (< 200 nm) to benefit from the enhanced permeability and retention (EPR) effect, which allows small particles to extravasate through the leaky vessels surrounding the tumor and accumulate inside the cancerous tissues. LFUS (at 20 kHz) applied in a pulsed mode triggered calcein release from the liposomes and the release increased with the increase in the US power density. Previous studies have also shown that LFUS can trigger drug release from the liposomes in a controlled manner^69–71^. LFUS can produce both thermal and mechanical effects; both may  contribute to enhancing drug release from the liposomes. However, the mechanical effects are more likely to be the main mechanism behind the triggered release. This is because liposomes are made of phospholipid walls that can lose integrity when heated above their transition temperature. When US was applied in our experiments, both types of liposomes released most of their content when the temperature did not exceed 31 °C, which is significantly lower than the transition temperature of DPPC, i.e., of 41.3 °C. This indicates that the mechanical effects in the form of cavitation, both stable and transient, are the likely driving forces behind drug release. Besides, the calculated MI values of 0.096, 0.115, and 0.121 for the three power densities used 6.2, 9 and 10 mW/cm^2^, respectively, are below the threshold required for the collapse cavitation to occur (MI = 0.3) indicating the occurrence of stable cavitation^59,61^.

Cavitation may cause a phenomenon known as “sonoporation”, which is the ability of US to modify cellular membranes' permeability by creating transient pores in the membrane. This is likely to be the mechanism leading to the triggered release reported here since liposome walls have a similar structure to cellular membranes (phospholipids bilayer). This is in agreement with previous studies which showed that cavitation-induced drug release from sonicated liposomes occured through pore formation rather than the destruction of the whole membrane^72,73^. We also showed that LFUS application was not toxic to the cells which confirms that sonoporation has no adverse effect on cell viability for the power density and frequency used in this study.

We have shown that SKBR3 (HER2 + cell line) cells showed higher calcein fluorescence intensity when incubated for 4 h with TRA-liposomes compared to the control liposomes. This was further supported with microscopic images of the cells. LFUS application further enhanced the fluorescent intensity of calcein. Also, when HER2 receptors were blocked with Herceptin molecules the intensity, of calcein fluorescent inside the SKBR3 cells was reduced. Similar calcein fluorescence intensity was observed in MDA-MB-231 (HER2- cell line) when incubated with both the control and immunoliposomes. These findings indicate that liposomes were taken up by the cells in different ways. Liposomes are made from phospholipids that are similar to those present in the biological membranes. Therefore, liposomes can transfer their load to the cytoplasm of the cells by fusing with the cellular membrane. This was seen when the control liposomes were incubated with SKBR3 cells. However, as previously discussed by Vogel et al.^74^, this process is slow and increases gradually with time. TRA-liposomes, on the other hand, are able to deliver their cargo to cells not only through membrane fusion but also through receptor-mediated endocytosis. This will allow the cellular uptake of a larger number of liposomes in a shorter period of time (4 h in this case) as was observed in Figs. 3 and 5. Four hours of incubation is sufficient for endocytosis/receptor recycling to take place. Previous studies have shown that endocytic recycling pathways can be fast (t_1/2_ = 1–5 min) or slow (t_1/2_ = 10–20 min)^75,76^. Application of US and the cavitation-mediated pore formation (sonoporation) on the phospholipids walls of the cancer cells and the walls of the liposomes, resulted in enhancing the uptake of the liposomes by the cells and calcein release from the liposomes as well. This was reflected in the enhancement of calcein fluorescence intensity inside the cancer cells.

A comparison of the performance of free DOX across both cell lines, with and without exposure to LFUS, suggested that LFUS exposure has a visible effect on the action and uptake of free DOX due to the sonoporation effect. This observation is in agreement with^77^. TRA conjugation had no significant effect on the cell viability of MDA-MB-231 when compared to the control but resulted in the significant inhibition of cell viability in SKBR3 cells which was further improved following exposure to US . These findings show that US is important in controlling drug release from the drug-loaded immunoliposomes. This makes the combination of immunoliposomes and US a promising targeted technique to treat HER2 + primary or secondary breast cancer cell lines. This can be achieved by injecting the patients with immunoliposomes encapsulating the drug, while a clinically approved ultrasonic probe focuses acoustic waves on the tumor, releasing the therapeutic agent and causing the formation of pores in the cell membrane of the diseased cells.

## Conclusion

The overexpression of HER2 receptors on the surface of some breast cancer cells provides a unique platform for HER2-targeted liposomes aiming to deliver their therapeutic to the diseased cells. In this study, we have successfully synthesized pegylated liposomes and decorated their surfaces with the monoclonal antibody Trastuzumab (TRA-liposomes). We also investigated the effect of applying LFUS to stimulate drug release in a controlled manner. Our in vitro results showed that the combination of Trastuzumab-conjugated liposomes and low-frequency ultrasound is a safe and effective technique in breast cancer treatment. This study is a proof of concept that can be further corroborated with future in vivo and clinical work to unlock the full potentials of this promising therapeutic technique.

## Supplementary Information

Supplementary information.
