# Supplementary material for: Ultrasound-triggered herceptin liposomes for breast cancer therapy
Source: Sci Rep. 2021 Apr 6;11:7545. doi: 10.1038/s41598-021-86860-5 (PMC8024284; doi:10.1038/s41598-021-86860-5)
Supplement: Supplementary file 1 — Supplementary information. [file 41598_2021_86860_MOESM1_ESM.docx]

Ultrasound-triggered Herceptin Liposomes for Breast Cancer Therapy

Amal Elamir,^a^ Saniha Ajith,^a^ Nour Al Sawaftah,^a^ Waad Abuwatfa,^a^ Debasmita Mukhopadhyay,^a^ Vinod Paul,^a^ Mohammad H. Al-Sayah,^b^ Nahid Awad,^a^ and Ghaleb A. Husseini^a,^*

^a^Department of Chemical Engineering, American University of Sharjah, Sharjah, United Arab Emirates

^b^Department of Biology, Chemistry and Environmental Sciences, American University of Sharjah, PO. Box 26666, Sharjah. UAE

*corresponding author, email: ghusseini@aus.edu

**Supplementary material**


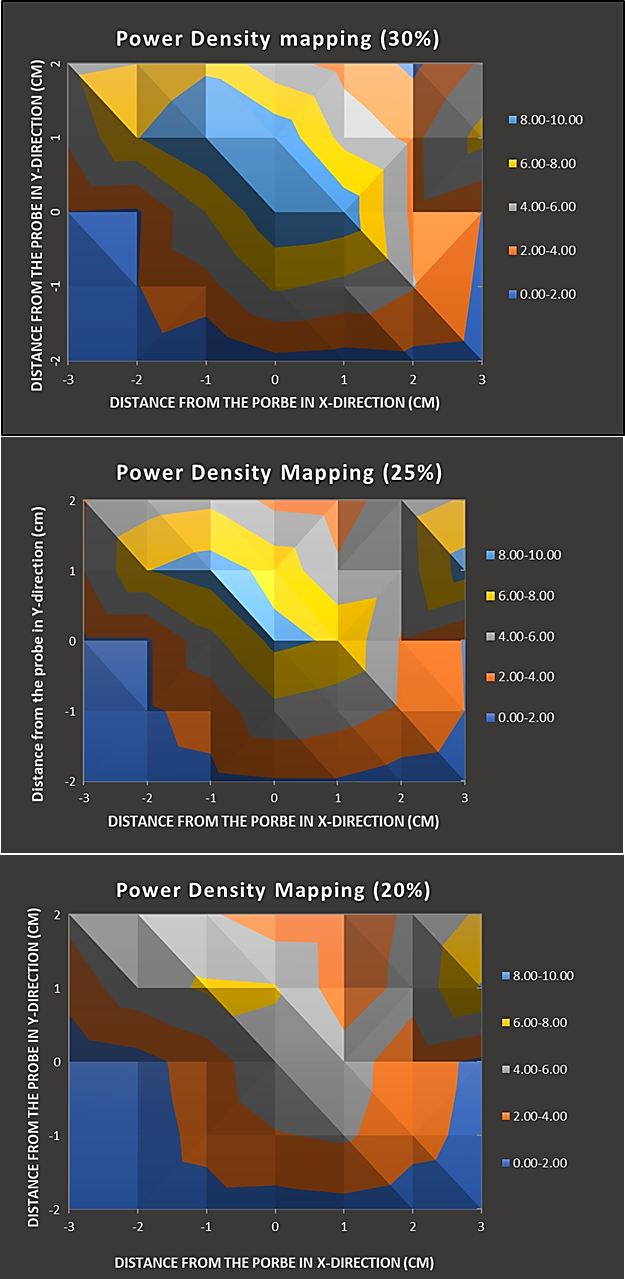


Figure 1. Power densities values measured with the hydrophone at various distances from the probe at 30%, 25% and 20% amplitude.

Table 1. Heat map summarizing p-values for the MDA-MB-231 cell line

|  | **Untreated** | **Untreated +LFUS** | | **DOX** | | **DOX+LFUS** | **Control-DOX** | **Control-DOX +LFUS** | **TRA-DOX** | **TRA-DOX+LFUS** |
| --- | --- | --- | --- | --- | --- | --- | --- | --- | --- | --- |
| **Untreated** | 1 | 9.58E-01 | | 7.64E-10 | | 4.17E-10 | 2.01E-07 | 5.76E-08 | 2.68E-04 | 2.54E-10 |
| **Untreated +LFUS** | 9.58E-01 | 1 | | 7.64E-10 | | 4.17E-10 | 2.01E-07 | 5.76E-08 | 2.68E-04 | 2.54E-10 |
| **DOX** | 7.64E-10 | 7.64E-10 | | 1 | | 1.55E-03 | 6.25E-07 | 2.03E-06 | 1.71E-04 | 1.03E-07 |
| **DOX+LFUS** | 4.17E-10 | 4.17E-10 | | 1.55E-03 | | 1 | 4.33E-07 | 1.23E-06 | 1.33E-04 | 4.81E-08 |
| **Control-DOX** | 2.01E-07 | 2.01E-07 | | 6.25E-07 | | 4.33E-07 | 1 | 1.60E-04 | 1.24E-01 | 8.44E-05 |
| **Control-DOX +LFUS** | 5.76E-08 | 5.76E-08 | | 2.03E-06 | | 1.23E-06 | 1.60E-04 | 1 | 5.48E-03 | 9.42E-02 |
| **TRA-DOX** | 2.68E-04 | 2.68E-04 | | 1.71E-04 | | 1.33E-04 | 1.24E-01 | 5.48E-03 | 1 | 6.78E-03 |
| **TRA-DOX+LFUS** | 2.54E-10 | 2.54E-10 | | 1.03E-07 | | 4.81E-08 | 8.44E-05 | 9.42E-02 | 6.78E-03 | 1 |
| **p =1** | **p > 0.05** | | **0.01< p <0.05** | | **0.001 < p < 0.01** | | **p < 0.001** |  |  |  |

Table 2. Heat map summarizing p-values for the SKBR3 cell line

|  | **Untreated** | | **Untreated +LFUS** | | **DOX** | | **DOX+LFUS** | **Control-DOX** | **Control-DOX +LFUS** | **TRA-DOX** | **TRA-DOX+LFUS** |
| --- | --- | --- | --- | --- | --- | --- | --- | --- | --- | --- | --- |
| **Untreated** | 1 | | 5.80E-01 | | 2.90E-09 | | 6.51E-08 | 6.98E-05 | 2.20E-07 | 4.48E-06 | 2.46E-10 |
| **Untreated +LFUS** | 5.80E-01 | | 1 | | 2.90E-09 | | 6.51E-08 | 6.98E-05 | 2.20E-07 | 4.48E-06 | 2.46E-10 |
| **DOX** | 2.90E-09 | | 2.90E-09 | | 1 | | 3.88E-03 | 3.13E-04 | 2.76E-04 | 6.03E-04 | 9.19E-05 |
| **DOX+LFUS** | 6.51E-08 | | 6.51E-08 | | 3.88E-03 | | 1 | 1.93E-04 | 1.55E-04 | 2.89E-04 | 1.59E-04 |
| **Control-DOX** | 6.98E-05 | | 6.98E-05 | | 3.13E-04 | | 1.93E-04 | 1 | 2.22E-03 | 1.23E-02 | 7.37E-04 |
| **Control-DOX +LFUS** | 2.20E-07 | | 2.20E-07 | | 2.76E-04 | | 1.55E-04 | 2.22E-03 | 1 | 4.12E-02 | 4.55E-03 |
| **sTRA-DOX** | 4.48E-06 | | 4.48E-06 | | 6.03E-04 | | 2.89E-04 | 1.23E-02 | 4.12E-02 | 1 | 3.18E-03 |
| **TRA-DOX+LFUS** | 2.46E-10 | | 2.46E-10 | | 9.19E-05 | | 1.59E-04 | 7.37E-04 | 4.55E-03 | 3.18E-03 | 1 |
| **p =1** | | **p > 0.05** | | **0.01< p <0.05** | | **0.001 < p < 0.01** | | **p < 0.001** |  |  |  |
